# Discharge to Temporary Accommodation From Acute Psychiatric Wards: A Service Evaluation (2023–2024)

**DOI:** 10.1192/bjo.2026.11579

**Published:** 2026-06-30

**Authors:** Joshua Wang, Sandeep Singh, Arnav Singh

**Affiliations:** 1 Leicestershire Partnership NHS Trust, Leicester, United Kingdom; 2 University Hospitals of Leicester NHS Trust, Leicester, United Kingdom

## Abstract

**Aims:**

Stable housing is a key determinant of mental health outcomes. Discharge from acute psychiatric inpatient services into temporary accommodation may reflect system pressures and can threaten recovery, continuity of care, and social stability. Despite this, limited data is available on discharge to temporary accommodation among psychiatric inpatients.

This service evaluation aims to describe patterns of discharge to temporary accommodation from acute adult inpatient psychiatric wards within one NHS Trust during the 2023–2024 financial year. We identified the types of temporary accommodation used, associated psychiatric diagnoses, and patient demographics.

**Methods:**

All patients discharged from acute adult inpatient wards to temporary accommodation between April 2023 and March 2024 were identified. We reviewed electronic patient records to identify primary diagnosis, discharge destination, and patient demographics. Temporary accommodation was grouped into categories including bed and breakfast (B&B) or hotel, supported living, care homes, and other temporary housing. Descriptive analysis examined distributions across accommodation type, diagnosis, and demographics.

**Results:**

During the 2023–2024 financial year, 146 patients were discharged from acute adult inpatient mental health wards to temporary accommodation. Psychotic disorders were the most common diagnosis (n=77), followed by personality disorders (n=38) and non-psychotic affective disorders (n=16). The remaining patients had diagnoses including dementia, anorexia nervosa, substance use disorders, or no recorded mental disorder.

Over half were discharged to B&B or hotel placements (n=77). Other discharge destinations included family or friends (n=27), interim housing (n=14), supported living (n=9), care homes (n=8), or no fixed abode (n=5). A small number were discharged to other temporary arrangements.

Patients with psychotic disorders formed ~50% those discharged to B&B or hotel accommodation (n=38), followed by patients with personality disorders (n=27). Of those discharged with no fixed abode, 3 had a diagnosis of personality disorder, 1 had a psychotic disorder, and 1 had a substance use disorder.

**Conclusion:**

Over half of patients discharged to temporary accommodation were placed in B&B or hotel settings, with psychotic disorders comprising ~50% of these patients. This highlights a notable amount of patients with severe mental illness being discharged to temporary, non-therapeutic accommodation. Patient discharge is a complex interplay of system pressures, clinical presentation and social support, yet stable housing is vital in holistic, patient-centred care. These results identify a need for further review of discharge pathways and raises the importance of the biopsychosocial model in understanding the relationship between mental health and social context.

